# Association of body mass index with long-term outcomes in older adults hospitalized for COVID-19: an observational study

**DOI:** 10.1038/s41598-024-58388-x

**Published:** 2024-03-29

**Authors:** Alain Putot, Charline Guyot, Patrick Manckoundia, Virginie Van Wymelbeke-Delannoy

**Affiliations:** 1 Service de Médecine Interne et Maladies Infectieuses, Hôpitaux du Pays du Mont Blanc, Sallanches, France; 2 Physiopathologie et Epidémiologie Cérébro-Cardiovasculaires (PEC2), Université de Bourgogne Franche Comté, Besançon, France; 3 Service de Médecine Interne Gériatrie, Pôle Personnes Agées, Centre Hospitalier Universitaire Dijon Bourgogne, Dijon, France; 4 Unité de Recherche Nutrition, Pôle Personnes Agées, Centre Hospitalier Universitaire Dijon Bourgogne, Dijon, France; 5 INSERM U1093 Cognition Action Plasticité, Université de Bourgogne Franche Comté, Besançon, France; 6 Centre des Sciences du Goût et de L’Alimentation, AgroSup Dijon, CNRS, INRAE, Université Bourgogne Franche-Comté, 21000 Dijon, France

**Keywords:** Viral infection, Geriatrics, Nutrition

## Abstract

Both underweight and obesity have been associated with poor prognosis in COVID-19. In an older populations of patients hospitalized for SARS-CoV-2 infection, we aimed to evaluate the association between body mass index (BMI) and short and long-term prognosis. Among 434 consecutive patients aged ≥ 70 years and hospitalized for suspected COVID-19 at a university hospital, 219 patients (median age of 83 years, 53% male) testing positive for COVID-19 and for whom BMI was recorded at admission, agreed to participate. Among them, 39 had a BMI < 20 kg/m^2^, 73 had a BMI between 20 and 24.9 kg/m^2^ and 107 had a BMI ≥ 25 kg/m^2^. After adjustment for confounders, BMI < 20 kg/m^2^ was associated with a higher risk of one-year mortality (hazard ratio (HR) [95% confidence interval]: 1.75 [1.00–3.05], *p* = 0.048), while BMI ≥ 25 kg/m^2^ was not (HR: 1.04 [0.64–1.69], *p* = 0.9). However, BMI was linearly correlated with both in-hospital acute respiratory failure (*p* = 0.02) and cardiovascular events (*p* = 0.07). In this cohort of older patients hospitalized for COVID-19, low BMI, rather than high BMI, appears as an independent risk factor for death after COVID-19. The pathophysiological patterns underlying this excess mortality remain to be elucidated.

## Introduction

Malnutrition is highly prevalent in hospitalized patients. This is especially true in the older population, in which rates of up to 30–50% are reported, indicating that this issue represents a major public health problem^1^. Indeed, malnutrition severely impairs prognosis in hospitalized patients^2,3^. Body mass index (BMI) is the most commonly used method to assess nutritional status in population studies. BMI and mortality are traditionally related in a J-shaped curve: the risk of death is increased in those with low or high BMI^4,5^.

During the COVID-19 pandemic, a similar J-shaped curve of correlation between BMI and mortality has been described. Indeed, both obesity and underweight have been shown to be significant independent risk factors for mortality from COVID-19^6,7^. Preexisting malnutrition is increasingly recognized as a risk factor of developing the disease and of severe presentation^8,9^, especially in older patients^10^. Conversely, there is also a very high risk of developing malnutrition during the course of COVID-19 with an overage risk estimated at nearly 50% in hospital^9^, with substantial impact on long-term prognosis^8,11^. Weight loss has been associated with longer disease duration and hospital stay^11,12^. Malnutrition screening and implementation of nutritional care have been associated with survival in observational studies^13–15^.

On the other hand, impact of obesity in the course of COVID-19 is still a matter of debate. Controversy exists regarding the prognostic effect of obesity in older population. At older ages, BMI associated with minimal mortality increases with age^16^ and excess weight may serve as a buffer against mortality^17^. Some studies have shown that obesity may reduce overall inpatient mortality risk, a phenomenon termed as the “obesity paradox”, evidenced both in the general population^18,19^ and in nursing-home residents^20^. This could be particularly true during COVID-19, as higher BMI is markedly associated with improved survival after acute infection^21^. Recent data suggest that obesity is not a risk factor for death in very old patients with COVID-19, and emphasize the role of underweight and malnutrition in geriatric patients with COVID-19^10^.

In this study, we aimed to evaluate the impact of BMI on mortality in older patients hospitalized for COVID-19, irrespective of age and comorbidities.

## Methods

### Population

In this retrospective, observational, monocentric study, we included all patients aged ≥ 70 years and hospitalized for COVID-19 (with a positive PCR test) in a French university hospital, regardless of the medical department, between 1 March and 31 May 2020, i.e. during the COVID-19 first wave in France. Patients for whom BMI was unavailable were not included. Data were extracted from medical records and anonymized before release to investigators.

This observational study was conducted in accordance with the Declaration of Helsinki and national standards. The protocol was approved by the Dijon University Hospital Ethics Committee. Each participant or his/her referee received an information letter prior to inclusion and was invited to oppose participation in the study if desired.

### Data collection

Socio-demographic data and comorbidities, evaluated through the Charlson Comorbidity Index (CCI)^22^, were collected at inclusion. Clinical presentation, including weight and BMI measured by the referring nurse at admission, and biological sampling including serum albumin, prealbumin and C-reactive protein rate sampled in the 48 h following admission, were also reported at admission. Serum levels of C-reactive protein, albumin, and prealbumin were measured using Cobas® immunoturbimetric method (Roche Diagnostics International Ltd, Rotkreuz, Switzerland).

In addition, we recorded in computerized medical files and collected retrospectively from the Hospital Database in-hospital events, including acute cardiovascular events (myocardial ischemia, myocardial infarction, acute heart failure, new atrial fibrillation, stroke, thromboembolism), acute respiratory failure (defined as arterial partial pressure of oxygen (Pa0_2_ < 60 mmHg), acute renal failure (defined as a twofold increase in plasmatic creatinine rate), delirium, as well as bacterial infection, anxiety-depression syndrome and loss of ambulation.

Vital status 30 days and one year after admission was obtained through the *Répertoire national d’identification des personnes physiques* (RNIPP), which is a French government database that records the vital status of all persons born or living in France. Baseline was defined as patient admission. Follow-up was done using computerized medical files for in-hospital events and through the RNIPP. There was no lost to follow-up, censoring occurred for patients still alive at one year. The proportional hazard assumption was tested graphically using a plot of the log cumulative hazard.

### Statistical analyses

Patients were compared according to the BMI group (< 20, 20–24.9 and ≥ 25 kg/m^2^). The thresholds were chosen according to the latest published international consensus: BMI < 20 kg/m^2^ defined severe malnutrition, as recommended by the last Global Leadership Initiative on Malnutrition (GLIM) guidelines^23^, while BMI ≥ 25 kg/m^2^ defined overweight, according to Word Health Organization (WHO) criteria.

Qualitative variables were expressed as numbers and percentages and compared with the Chi-2 or Fischer tests, as appropriate. Continuous variables were expressed as median and interquartile range and compared with the Mann Whitney U test.

Kaplan–Meier curves and log-rank tests were used to compare survival times according to the BMI group.

Factors associated with mortality at 30 days and one year were analyzed with a multivariate analysis by a Cox model integrating predetermined clinically relevant variables: age, sex and CCI. Adjusted hazard ratios (aHR) were thus evaluated according to BMI groups. To evaluate the prognostic impact of obesity vs. overweight, BMI ≥ 25 kg/m^2^ group was dichotomized in overweight (BMI 25–29.9 kg/m^2^) and obese patients groups (BMI ≥ 30 kg/m^2^) in multivariate models, according to WHO criteria.

Prognostic value of nutritional biomarkers (serum albumin and prealbumin) was evaluated using C-statistics.

The threshold for significance (*p*) was set to 5%. SPSS version 12.0.1 (IBM, Armonk, NY, USA) was used for all statistical tests.

### Institutional review board statement

The study was conducted in accordance with the Declaration of Helsinki. The protocol was approved by the Dijon University Hospital Ethics Committee.

### Informed consent

Each participant or his/her referee received an information letter and was invited to express his/her opposition to participation in the study. However, due to the retrospective nature of the study and the use of anonymized data, Dijon University Hospital Committee waived the need of obtaining informed consent.

## Results

### Population

**Figure 1:**
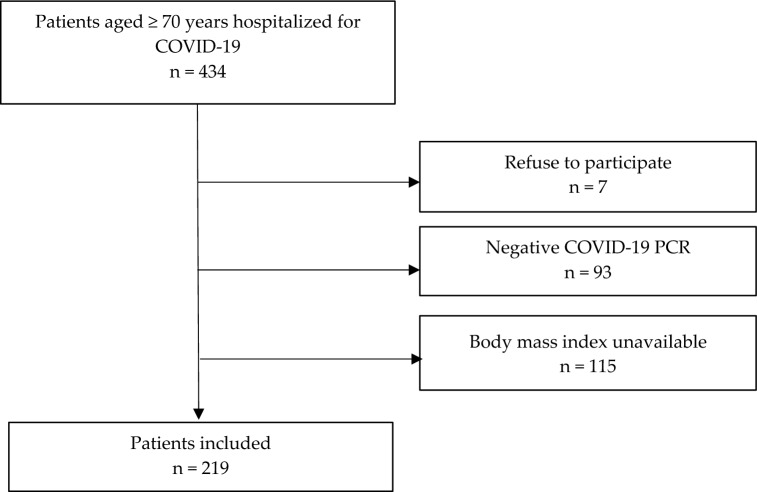
Flow chart.

Among 434 consecutive patients aged ≥ 70 years, hospitalized for COVID-19 during the first epidemic wave in France, 219 with positive COVID-19 PCR and available BMI agreed to participate (Fig. 1).

Socio-demographic characteristics, clinical presentation and biological parameters at admission are described in Table 1. Median age was 83 years (range 75–88, men 53%). Thirty-nine patients (18%) had a BMI < 20 kg/m^2^, 73 (33%) had a BMI between 20 and 24.9 kg/m^2^ and 107 (49%) had a BMI ≥ 25 kg/m^2^. BMI was inversely correlated with age (*p* = 0.005) and frequency of neurocognitive disorders (*p* = 0.03), was associated with more frequent diabetes (*p* = 0.02), whereas comorbidities burden evaluated by CCI did not differ between the three groups (*p* = 0.02). Neither clinical presentation, nor biological parameters at admission significantly differ between the BMI groups.

**Table 1:** Characteristics at admission (n (%) or median [interquartile range]).

	All patients n = 219	BMI < 20 n = 39	BMI 20–24.9 n = 73	BMI ≥ 25 n = 107	*P*
Demographics					
Age (years)	83 [75–88]	85 [80–91]	85 [78–89]	80 [74–86]	0.005
Men	117 (53)	16 (41)	38 (52)	63 (59)	0.1
Comorbidities					
Myocardial Infarction	29 (13)	6 (15)	9 (12)	14 (13)	0.9
Heart failure	103 (47)	19 (49)	32 (44)	52 (49)	0.8
Peripheral artery disease	46 (21)	4 (10)	17 (23)	25 (24)	0.2
Stroke	57 (26)	9 (23)	20 (27)	28 (26)	0.9
Diabetes	66 (30)	11 (28)	14 (19)	41 (39)	0.02
Chronic respiratory disease	37 (17)	5 (13)	11 (15)	21 (20)	0.5
Chronic kidney disease	54 (25)	11 (28)	21 (29)	22 (21)	0.4
Chronic liver disease	12 (5)	1 (3)	3 (4)	8 (7)	0.4
Cognitive disorders	92 (42)	23 (59)	32 (44)	37 (35)	0.03
Active Neoplasia	42 (19)	5 (13)	20 (27)	17 (16)	0.09
Charlson Comorbidity index	3 [2–5]	3 [1–5]	3 [1–5]	3 [2–4]	1
Clinical presentation					
Weight (kg)	68 [58–81]	48 [43–55]	62 [57–65]	81 [74–90]	< 0.001
BMI (kg/m^2^)	25 [22–29]	19 [18–20]	23 [22–24]	29 [27–33]	< 0.001
Heart rate (/min)	79 [70–90]	82 [67–89]	77 [71–92]	79 [70–93]	0.8
Systolic blood pressure (mmHg)	130 [117–145]	125 [117–139]	128 [116–148]	134 [119–150]	0.2
Diastolic blood pressure (mmHg)	68 [60–80]	60 [56–78]	68 [60–80]	69 [64–81]	0.05
Temperature (°C)	37.2 [36.7–37.8]	37.0 [36.5–37.8]	37.1 [36.7–37.8]	37.3 [36.7–37.8]	0.6
Respiratory rate (/min)	22 [18–26]	22 [17–27]	20 [17–24]	24 [19–28]	0.3
Oxygen Saturation (%)	95 [93–97]	96 [94–97]	95 [93–97]	95 [93–97]	0.4
Oxygen supply	28 (13)	6 (15)	8 (11)	14 (13)	0.8
Biological markers					
C-reactive protein (mg/L)	53 [17–131]	44 [14–82]	54 [16–125]	55 [20–154]	0.3
Albumin (g/L) (n = 149)	26 [23–30]	25 [23–30]	26 [23–30]	27 [24–31]	0.5
Prealbumin (g/L) (n = 65)	0.12 [0.09–0.16]	0.13 [0.09–0.19]	0.09 [0.07–0.15]	0.12 [0.10–0.18]	0.08

*BMI* Body Mass Index (kg/m^2^).

### Outcomes

In-hospital events following admission for COVID-19 and the main outcomes are reported in Table 2. Median hospital stay was 15 [8–33] days. Acute cardiovascular events occurred in half of patients, and their frequency tended to increase with BMI (*p* = 0.07). Acute respiratory failure occurred 93% of patients overall, reaching 97% in patients with BMI ≥ 25 kg/m^2^ (*p* = 0.02). In-hospital mortality tended to be higher in patients with BMI < 20 kg/m^2^ (41% vs. 25% for BMI between 20 – 25 kg/m^2^ and 27% for BMI ≥ 25 kg/m^2^, *p* = 0.2) and was significantly higher at one-year (61% vs. 38% and 36% respectively, *p* = 0.02).

**Table 2:** In-hospital events and outcomes (n (%) or median [interquartile range]).

Variables	All patients	BMI < 20	BMI 20 – 24.9	BMI ≥ 25	*P*
	n = 219	n = 39	n = 73	n = 107	
In-hospital events					
Cardiovascular events	114 (52)	16 (41)	34 (47)	64 (60)	0.07
Myocardial ischemia	64 (29)	7 (18)	19 (26)	38 (35)	0.09
Myocardial infarction	6 (3)	0	1 (1)	5 (5)	0.2
Acute heart failure	32 (15)	5 (13)	7 (10)	20 (19)	0.2
Thromboembolism	10 (5)	1 (3)	2 (3)	7 (6)	0.4
Stroke	7 (3)	1 (3)	1 (1)	5 (5)	0.5
New atrial fibrillation	31 (14)	3 (8)	13 (18)	15 (14)	0.3
Acute respiratory failure	203 (93)	33 (85)	66 (90)	104 (97)	0.02
Acute renal failure	47 (21)	3 (8)	17 (23)	27 (25)	0.07
Alertness disorders	46 (21)	4 (10)	16 (22)	26 (24)	0.2
Delirium	27 (12)	5 (13)	9 (12)	13 (12)	1
Bacterial infection	49 (22)	5 (13)	19 (26)	25 (23)	0.3
Anxiety-depression	40 (18)	10 (26)	13 (18)	17 (16)	0.4
Loss of ambulation	21 (10)	7 (18)	7 (10)	7 (6)	0.2
Outcomes					
Hospital stay (days)	15 [8–33]	20 [8–49]	14 [7–35]	14 [9–29]	0.5
Hospital death	63 (29)	16 (41)	18 (25)	29 (27)	0.2
Death at 30 days	60 (27)	15 (38)	17 (23)	28 (26)	0.2
Death at one year	91 (42)	24 (61)	28 (38)	39 (36)	0.02

*BMI* Body Mass Index (kg/m^2^).

**Figure 2:**
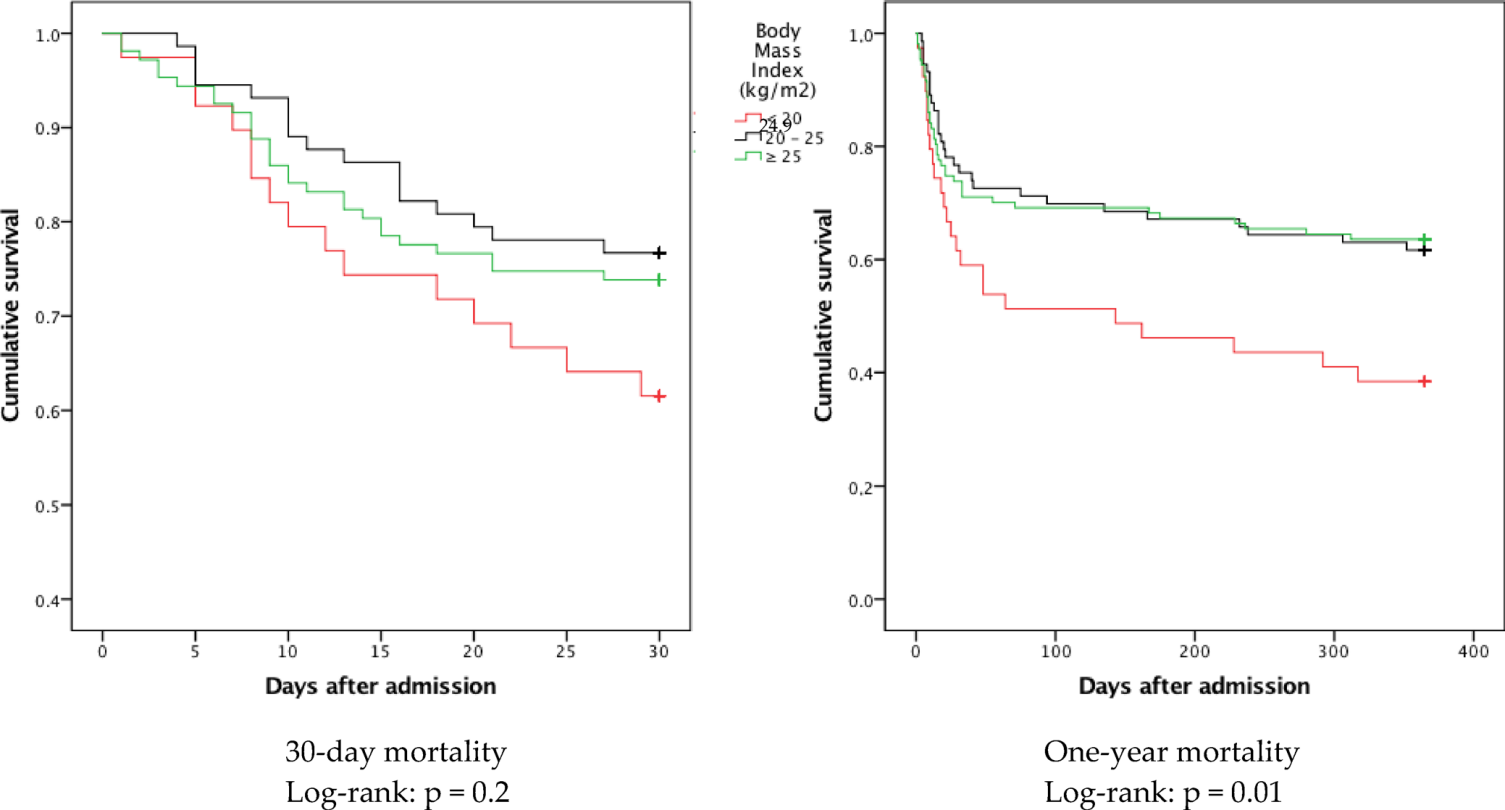
Kaplan–Meier curves for 30-day (left) and one-year (right) mortality after hospitalization for COVID-19 according to the body mass index.

30-day and one-year Kaplan–Meier Curves found similar survival profiles for patients with normal or high BMI, whereas patients with BMI < 20 kg/m^2^ had a significantly worse long-term prognosis (Fig. 2).

After adjustment on age, sex and CCI, BMI < 20 kg/m^2^ remained associated with a nearly doubled 30-day mortality (aHR (95% confidence interval): 1.92 (0.92–3.99), *p* = 0.08) and at one-year mortality (1.86 (1.05–3.29), *p* = 0.03). Overweight (BMI ≥ 25 kg/m^2^) was associated with a non-significant excess risk of death at 30 days (aHR: 1.45 (0.79–2.67), *p* = 0.2), but not at one year (aHR: 1.04 (0.64–1.69), p = 0.9).

**Figure 3:**
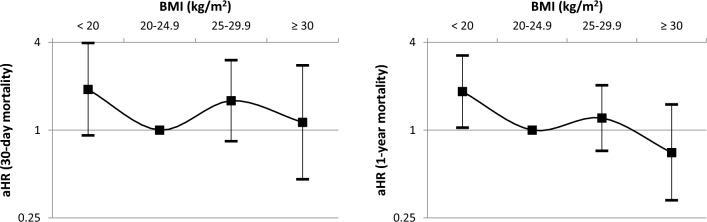
Adjusted hazard ratio (aHR) for 30-day (left) and one-year (right) mortality according to the body mass index (BMI, reference group: BMI 20–24.9 kg/m^2^).

When further dichotomizing BMI ≥ 25 group in overweight (BMI 25–29.9 kg/m^2^) and obese (BMI ≥ 30 kg/m^2^) patients, obese patients (n = 37) tended to present a lower mortality rate than overweight patients after adjustment on confounders (Fig. 3), although this difference did not reach significance.

### Biomarkers

Nutritional biomarkers were not associated with short or long-term prognosis. C-statistics (95% Confidence interval) for serum albumin were 0.55 (0.45–0.65), *p* = 0.4 and 0.52 (0.43–0.61), *p* = 0.7 for predicting 30-day and one-year survival, respectively.

C-statistics for serum pre-albumin were 0.47 (0.31–0.62), *p* = 0.7 and 0.52 (0.38–0.66), *p* = 0.8 for predicting 30-day and one-year survival, respectively.

C-reactive protein did not better predict prognosis: C-statistics for C-reactive protein were 0.57 (0.49–0.65), *p* = 0.1 and 0.55 (0.47–0.62), *p* = 0.3 for predicting 30-day and one-year survival, respectively.

## Discussion

In a population of older patients hospitalized for COVID-19, we aimed to investigate the prognostic burden of BMI, irrespective of comorbidities and age.

A first result of this study is the great frequency of both malnutrition and overweight in this population: nearly 18% had a BMI < 20 kg/m^2^, classified as severe malnutrition according to the last GLIM criteria^23^. Overweight was even more frequent: nearly half of patients were overweight and among them 17% were obese. These findings are in accordance with previous reports in very old populations as concerns the prevalence of malnutrition, but highlight clearly that the proportion of overweight and obese patients was higher among those admitted for COVID-19 as compared with general older population^20^. In a prospective Survey across 28 European countries among 1936 European individuals aged 50 and older, over 75% of COVID-19 related hospitalization were overweight^24^. Compared to non-obese patients, obese individuals have an increased risk of COVID-19^25^, and a doubled risk of hospitalization^26^. Obesity alone is responsible for nearly a third of all COVID-19 hospitalizations^27^.

Our results showed that older COVID-19 patients with a BMI under 20 kg/m^2^ had a higher risk of death than those in the normal BMI group, but we found no excess risk for overweight and obesity, despite a clear increase in in-hospital cardiovascular and respiratory events. Similar findings were found in older Swedish inpatients. After adjusting for age, sex, comorbidity, polypharmacy and frailty, underweight doubled the risk of in-hospital mortality, while overweight and obesity were not associated with in-hospital mortality^10^. Compared with BMI, visceral adipose tissue and intrathoracic fat are better predictors of COVID-19 severity and indicate the need for hospitalization in intensive care unit and invasive mechanical ventilation^28^. Some reports found that patients in the underweight, normal and grade 3 obesity (BMI ≥ 40 kg/m^2^*)* categories have a higher risk of COVID-19 related mortality, compared to those with BMI between 25 and 40 kg/m^2^, suggesting a potential protective effect of overweight and non-severe obesity in COVID-19 (obesity paradox)^21,29–32^. Such association between higher BMI and survival has already been highlighted in older nursing-home residents without COVID-19^20,33,34^. Our data do not confirm such findings in older hospitalized patients. Conversely, other authors found that obese patients are at higher risk of mortality in COVID-19 after adjustment for confounders^30,35–37^. Finally, the variability of mortality rate according to the BMI may be explained by confounding factors, such as younger age, fewer comorbidities and less severe organ failures^35,38^. Among 55,299 American patients testing positive for COVID-19, obesity alone did not significantly increase the risk of severe clinical outcomes. Obesity-related comorbidities (hypertension, diabetes), on the other hand, resulted in a significantly higher risk of outcomes^39^.

In a large cohort study of 6.9 million people in England in which 3% of the patients were underweight, a J-shaped association between BMI and death due to COVID-19 was identified, indicating an increased risk of death in people with BMI ≤ 20 kg/m^2^ after adjusting for confounders, which supports our findings^6^. Other studies specifically dedicated to older patients found similar results^10,31^. Practically all forms of immunity are affected by protein-energy malnutrition, but non-specific defenses and cell-mediated immunity are most severely affected^40^. The association between low BMI and short-term outcome could be due to an increased sepsis-related mortality linked to immunosenescence. Indeed, the immune system of older people is increasingly recognised as depending on nutritional status^41^. However, in our report, low BMI was not associated with a higher risk of bacterial infection during hospital stay, and respiratory failure and cardiovascular events were even less frequent. Underweight may be a marker of unintended weight loss in some patients related to poor underlying health and undiagnosed comorbid conditions rather than the direct cause of poor outcomes. We hypothesize that low BMI is a sign of frailty predisposing to long-term mortality. This corroborates the conclusions of a recent meta-analysis by the Global BMI Mortality Collaboration^5^.

We found surprising results concerning the absence of significant predictive value of serum albumin and prealbumin, considering that these nutritional biomarkers are known to be associated with survival in older patients with acute infection^42^, and especially after pneumonia^43,44^. Previous reports highlight poor yet significant correlation between low albumin and prealbumin rates and mortality in patients with COVID-19^45,46^. However, these biomarkers could have better prognostic value at the third day of hospital stay rather than at admission as in our study^45^, as a marker of the intensity of the inflammatory response.

This study has several limitations. First, the interpretation of these results is limited by its retrospective design. A large number of very old patients hospitalized for COVID-19 could not be weighed on admission, but this is due to the real-life conditions during the first wave of the COVID-19 pandemic. In the emergency context, with saturation of the care system, priority was given to the most urgent care. Secondly, for the same reasons, we were unable to evaluate the weight trajectory, and any weight loss before and after the COVID-19 episode, as well as food intake. Our report is not exhaustive of all malnourished patients because all phenotypic criteria for malnutrition (non-volitional weight loss, reduced muscle mass) were not collected^23^. Thirdly, although the prognostic impact of BMI was evaluated after adjustment on age, sex and comorbidities, multivariate models did not include several potential confounders such as socioeconomic conditions and initial severity parameters of infection, since prognostic scores at admission were not reported. However, clinical presentation at admission did not significantly differ between the BMI groups. Fourthly, the monocentric design and the small number of included patients are limitations, and it remains possible that some associations between BMI groups and mortality could not be shown due to lack of power. Especially, multivariable models failed to highlight a statistically significant difference in mortality at 30 days between groups. We believe however that the doubling of mortality at both 30 days and one year in the low BMI group remains clinically significant, despite the lack of power. Moreover, this study concerns older French inpatients, predominantly white, hospitalized for COVID-19. Whether these results can be extrapolate to other population with COVID-19, especially to outpatients, remains unknown. Finally, at long term, it remains difficult to discern the true burden of COVID-19 in this old frail population among multiple comorbidities, including the malnutrition and its causes. Indeed, the overall mortality at one year did not significantly differ from that observed in an unselected population form our geriatric unit (42 vs 37%, p = 0.2)^47^.

In conclusion, underweight (BMI < 20 kg/m^2^), unlike overweight (BMI ≥ 25 kg/m^2^), appears as an independent risk factor for death at one year in older patients hospitalized for COVID-19. However, in-hospital respiratory failure and cardiovascular events increase linearly with BMI. The pathophysiological patterns underlying this excess mortality in low BMI patients remain to be elucidated.

## Data Availability

Data supporting reported results are available from the corresponding author on reasonable request.
